# Dipping contacts – a novel type of contact site at the interface between membraneless organelles and membranes

**DOI:** 10.1242/jcs.261413

**Published:** 2023-12-27

**Authors:** Christian Hoffmann, Dragomir Milovanovic

**Affiliations:** ^1^ Laboratory of Molecular Neuroscience, German Center for Neurodegenerative Diseases (DZNE), 10117 Berlin, Germany; ^2^ Whitman Center, Marine Biological Laboratory, Woods Hole, MA 02543, USA; ^3^ National Center for X-ray Tomography, Advanced Light Source, Berkeley, CA 94720, USA; ^4^ Einstein Center for Neuroscience, Charité-Universitätsmedizin Berlin, Freie Universität Berlin, Humboldt-Universität Berlin and Berlin Institute of Health, 10117 Berlin, Germany

**Keywords:** Dipping contacts, Liquid–liquid phase separation, Membranes, Neurodegenerative diseases, Synapse

## Abstract

Liquid–liquid phase separation is a major mechanism for organizing macromolecules, particularly proteins with intrinsically disordered regions, in compartments not limited by a membrane or a scaffold. The cell can therefore be perceived as a complex emulsion containing many of these membraneless organelles, also referred to as biomolecular condensates, together with numerous membrane-bound organelles. It is currently unclear how such a complex concoction operates to allow for intracellular trafficking, signaling and metabolic processes to occur with high spatiotemporal precision. Based on experimental observations of synaptic vesicle condensates – a membraneless organelle that is in fact packed with membranes – we present here the framework of dipping contacts: a novel type of contact site between membraneless organelles and membranes. In this Hypothesis, we propose that our framework of dipping contacts can serve as a foundation to investigate the interface that couples the diffusion and material properties of condensates to biochemical processes occurring in membranes. The identity and regulation of this interface is especially critical in the case of neurodegenerative diseases, where aberrant inclusions of misfolded proteins and damaged organelles underlie cellular pathology.

## Teaser

**Summary:** We employ the framework of dipping contacts – a novel type of contact site between membraneless organelles and membranes – to discuss the fate of condensates upon their interaction with membranes.

## Introduction

Liquid-liquid phase separation (LLPS) is a thermodynamic process in which macromolecules demix from the surrounding emulsion into distinct territories (Banani et al., 2017; Choi et al., 2020), forming biomolecular condensates. Structures both in the cytoplasm and nucleoplasm have been shown to assemble via LLPS, and these structures range in size from several nanometers (for example, transcription hubs) to the micrometer scale (for example, stress granules), pointing to LLPS being a scale-independent process (Lyon et al., 2021). As condensates are not limited by a membrane or a scaffold, they are often referred to as membraneless organelles (see Box 1). Classically, a cell is perceived as a complex network of membrane-bound organelles that are remodeled in response to signaling, allowing for cell growth, differentiation and response to a changing environment. Similarly, condensates are typically conveyed and pictured as separate entities in the cytoplasm. If a cell is a complex concoction of membraneless and membrane-bound organelles, then a critical question arises: what determines the fate of membraneless and membrane-bound compartments upon their initial interaction?

**Box 1. Concepts and Glossary**

To address a broad cell biology community, we provide here our working definitions that pertain to the field of condensate biology. Note that some aspects have been simplified; for the stringent and comprehensive definitions of the (bio)chemical aspects of biomolecular phase separation, we suggest an excellent recent review (Pappu et al., 2023).

**Liquid–liquid phase separation**

In cell biology, LLPS is a process where macromolecules condense from the surrounding environment into a dense phase that is not surrounded by a lipid membrane or protein scaffold.

**Saturation concentration**

The minimal concentration of a given macromolecule at which phase separation is triggered is referred to as the saturation concentration (*C*_sat_). Note that *C*_sat_ will depend on the local conditions in the cell. The ionic strength (which can be affected by, for example, the presence of small metabolites or polyelectrolytes), the presence of posttranslational (for proteins) or posttranscriptional (for RNA) modifications, and the presence of interacting and/or binding partners can all alter *C*_sat_ for a given macromolecule.

**Partition coefficient**

The partition coefficient is the ratio of molecular concentrations in a dense phase and a dilute phase. It describes the enrichment of a given macromolecule within a dense phase in comparison to its concentration in the surrounding medium.

**Emulsions**

Here, we consider an emulsion to be a mixture of two or more co-existing condensates that are normally immiscible as a result of LLPS.

**Membrane wetting**

The process of membrane wetting is a thermodynamically complex interplay between the interfacial (surface) tension of condensates (as ‘liquids’ try to minimize their surface tension, often relaxing in round structures) and the strength of adsorption of the condensate to the membrane (de Gennes et al., 2004). Condensates at surfaces can adopt different shapes depending on the interaction strength. They can remain spherical (no wetting); spread slightly on the surface (partial wetting); or spread completely, wetting the whole surface (complete wetting).

**Fig. 1.**
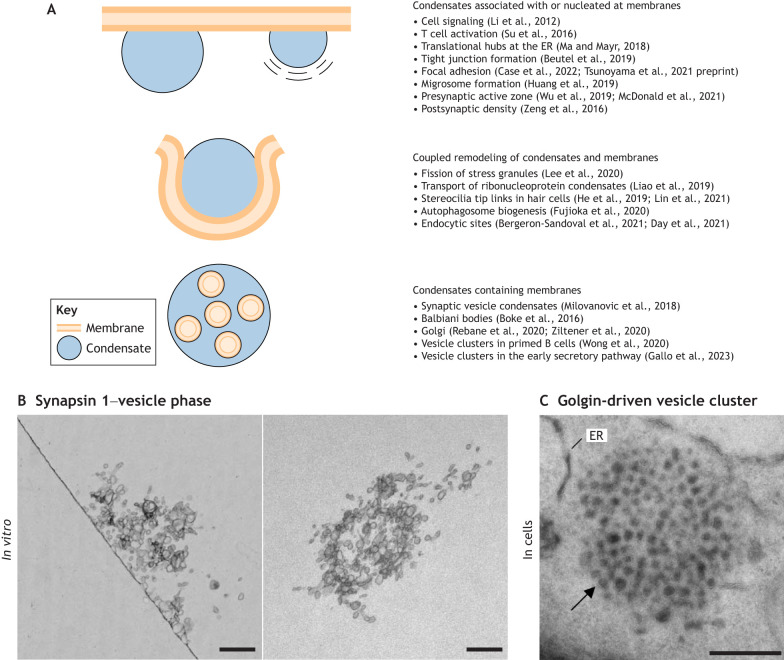
**Interactions of membraneless organelles with membrane-bound organelles.** (A) Three main types of interactions between membraneless organelles and membranes have been described in the literature: condensates associated with a membrane (top), coupled remodeling of condensates and membranes (middle), and membranes inside condensates (bottom). (B) EM images of reconstituted synapsin 1–lipid vesicle condensates. The left panel shows a section perpendicular to the liposome–glass interface, and the right panel shows a section parallel to the glass surface but above the glass. Modified, with permission, from Milovanovic et al. (2018). Reprinted with permission from AAAS. This image is not published under the terms of the CC-BY license of this article. For permission to reuse, please see Milovanovic et al. (2018). (C) EM image of a yeast cell ectopically expressing the N-terminal region of the golgin GMAP-210 (also known as TRIP11), showing the accumulation of secretory vesicles in distinct mesoscale territories (arrow). Image used with permission of Rockefeller University Press, from Pranke et al. (2011); permission conveyed through Copyright Clearance Center, Inc. This image is not published under the terms of the CC-BY license of this article. For permission to reuse, please see Pranke et al. (2011). Scale bars: 250 nm.

Thus far, three main types of association between biomolecular condensates and membranes have been described (Fig. 1). First, the condensates can adhere to juxtaposed membranes (Li et al., 2012; Su et al., 2016; Zeng et al., 2016; Ma and Mayr, 2018; Beutel et al., 2019; Tsunoyama et al., 2021 preprint; Case et al., 2019; Huang et al., 2019; Wu et al., 2019; McDonald et al., 2021). Second, membrane-bound organelles can sequester condensates for trafficking across the cell; act as a signaling platform for vesicle budding or autophagosome formation, where the membrane remodeling is facilitated by the condensates; and triage functional condensates from dysfunctional inclusions (Liao et al., 2019; He et al., 2019; Lin et al., 2021; Lee et al., 2020; Fujioka et al., 2020; Bergeron-Sandoval et al., 2021; Day et al., 2021). Third, the membrane-bound organelles can be an integral part of membraneless condensates (Milovanovic et al., 2018; Gallo et al., 2023; Ziltener et al., 2020; Wong et al., 2020).

Several lines of experimental evidence strongly suggest that membranes play a crucial role in the spatiotemporal control of initial condensate nucleation. In the case of signaling molecules on the plasma membrane, the condensates nucleate directly on the membrane, either because a critical receptor protein scaffold is only found on the membrane (Banjade and Rosen, 2014; Su et al., 2016) or because the membranes can locally concentrate cytosolic proteins on the membrane, allowing for phase separation far below the bulk saturation concentration (Beutel et al., 2019; Honerkamp-Smith et al., 2009). Similar condensate nucleation events on membranes could also occur on other membrane-bound organelles, such as the endoplasmic reticulum and Golgi (Ma and Mayr, 2018; Ziltener et al., 2020). However, this is distinct from the scenario where a condensate forms independently in the cytosol and then encounters a membrane (Liao et al., 2019).

In the case of condensates that associate with or nucleate at membranes (Fig. 1A, top), the contacts between the condensate and the membrane are crucial for their particular biological function. For instance, the molecules within a condensate are several orders of magnitude more concentrated than in the surrounding region and can have increased enzymatic activity in the presence of a substrate (Peeples and Rosen, 2021) or a longer dwell time than outside the condensate (Case et al., 2019). Such condensates can functionally regulate protein–lipid domains in the membrane (Shelby et al., 2023; Wang et al., 2023), allowing for the formation of so-called ‘AND’ gate systems in which both signals in the cytoplasm and signals in the membrane need to occur concomitantly to trigger the downstream function (Tsunoyama et al., 2021 preprint). Such a scenario has been described for adhesion and cell motility, when lipids and receptors in the plasma membrane act as molecular switches triggering the formation of condensates at the membrane interface that facilitate the sequestering of downstream effector molecules (Ramella et al., 2022; Sun et al., 2023 preprint).

Another notable functional output of these contacts is that biomolecular condensates might induce a force that remodels the membrane (Fig. 1A, middle), as has been proposed to occur in endocytosis (Day et al., 2021; Bergeron-Sandoval et al., 2021) and the generation of autophagosomes (Agudo-Canalejo et al., 2021). Here, the difference in surface tension between condensate–cytoplasm and condensate–membrane interfaces could generate capillary forces that are sufficiently large to remodel the lipid bilayer (Bergeron-Sandoval et al., 2021; Gouveia et al., 2022; Mangiarotti et al., 2023). Indeed, successful clathrin-mediated endocytosis occurs only when initiation complexes – such as Eps15, FCHo1 and FCHo2 – form liquid-like assemblies at the membrane surface (Day et al., 2021; Kozak and Kaksonen, 2022); this induces steric pressure and bends the membrane into a spherical vesicle ready for uptake (Houser et al., 2022; Mund et al., 2023).

Autophagy is a major cellular degradation machinery that removes unwanted and/or damaged cellular components (Bauer et al., 2023; Yamamoto et al., 2023). It is a catabolic and signaling process in eukaryotic cells that involves sensing pathways, cargo selection, and major membrane remodeling that ultimately links sequestered cargoes to the growing autophagosomes and lysosomes. In this complex cellular process, LLPS plays an important role at multiple steps (Noda et al., 2020), including regulation of the transcription factors responsible for autophagosome and lysosome biogenesis (Chen et al., 2020; Wang et al., 2022), selection of cargos (Zhang et al., 2018; Sun et al., 2018; Zaffagnini et al., 2018; Yamasaki et al., 2020) and the actual remodeling of autophagosome membranes (Fujioka et al., 2020). In particular, multiple interactions between cargo containing p62 (also known as SQSTM1) and the growing autophagosome membrane form in a piecemeal fashion, wetting the membrane with lipidated Atg8 proteins (which are key components of the autophagy machinery) and thereby increasing the overall avidity and promoting growth of the autophagosome (Agudo-Canalejo et al., 2021; Maruyama et al., 2021; for basic concepts of membrane wetting see Box 1).

Finally, condensates, such as the clusters of synaptic vesicles (SVs) in nerve terminals or the Golgi stacks, can contain membrane-bound organelles as an integral part (Fig. 1A, bottom), allowing for functional mobilization and release of these membranous organelles upon appropriate signal activation. Such reversible mobilization and release could span from seconds (for example, SV dynamics; Milovanovic et al., 2018) over minutes (for example, vesicle trafficking across Golgi stacks; Ziltener et al., 2020) to even years (for example, Balbiani bodies; Boke et al., 2016).

**Fig. 2.**
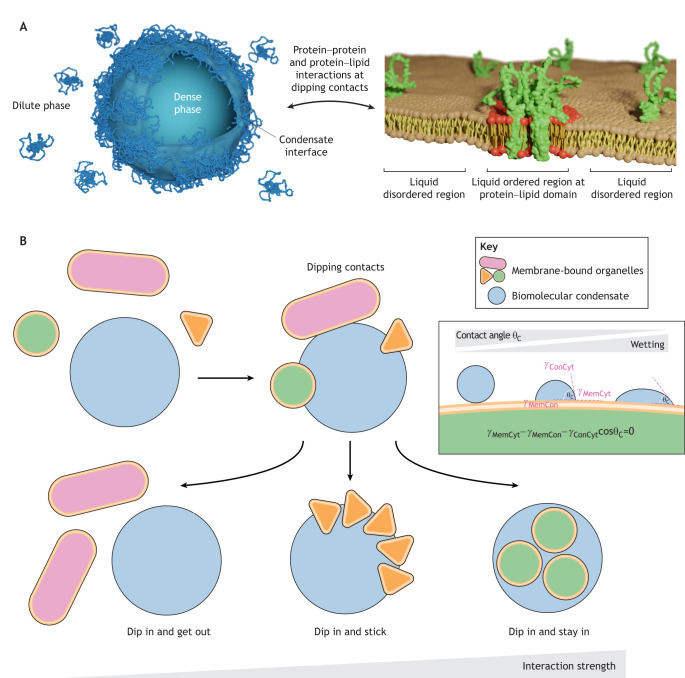
**Dipping contacts as a mechanism for the interaction between membraneless and membrane-bound organelles.** (A) Schematic illustration of an ensemble of proteins at the interface of a condensate engaging with protein–lipid domains in a membrane. Left: schematic of a condensate with the surface molecules highlighted. Right: schematic of a membrane containing protein oligomers that form a liquid ordered phase within a bilayer. AlphaFold predictions for the structures of synapsin 1 (AF-P17600) and synaptophysin (AF-P08247) are used to illustrate condensate and membrane proteins, respectively (Jumper et al., 2021). (B) Membrane-bound organelles interact transiently with a biomolecular condensate, resulting in a dipping contact. The dipping is followed either by repulsion, if no interactions occur (dip in and get out), by the accumulation of membrane at the interface of a condensate, if the membrane stabilizes it (dip in and stick), or by engulfment, if sufficient interactions occur between the membrane and the condensate (dip in and stay in). The inset depicts the wetting scenarios between a condensate and a membrane, with the wettability of condensate to membrane being quantified as the inverse of the contact angle, which is the angle between a tangent to the condensate interface and the membrane interface at their contact. γ_ConCyt_, condensate–cytosol interfacial energy; γ_MemCon_, membrane–condensate interfacial energy; γ_MemCyt_, membrane–cytosol interfacial energy.

All of these examples highlight the effects of membraneless condensates that result from their associations with membranes (for a review, see Snead and Gladfelter, 2019). In many scenarios, the formation of condensates depends on membranes, as the membranes provide a platform to recruit and locally enrich condensate components. However, at present no framework exists to explain what determines the fate of condensates after the initial contact occurs between the already formed membraneless and membrane-bound organelles (Fig. 2A). Here, we propose that these contact sites between condensates and membranes – termed ‘dipping contacts’ – play a crucial role in determining the downstream cascade of condensate–membrane assemblies. We put forward the synaptic bouton as a particularly suitable system for investigating condensate–membrane interactions in the context of physiology and membrane trafficking. Finally, we suggest that the build-up of intracellular inclusions identified in several neurodegenerative diseases might be a consequence of faulty dipping contacts.

## A proposed framework for the interface between membraneless organelles and membranes

Our proposed framework (summarized in Fig. 2B) is that the membrane-bound organelle dips transiently into the liquid phase, resulting in a dipping contact. A dipping contact between a membrane-bound organelle and a liquid condensate can result in various wetting scenarios depending on the relative strength of the interfacial tension between the membrane, the condensate and the cytosol. The wettability of the condensate on the membrane is quantified by the contact angle (see inset in Fig. 2B) and can be modified by proteins at the interface of condensates (Farag et al., 2022) or by membrane composition (Rouches et al., 2021; Zhao et al., 2021). In the case of strong wetting, which is characterized by a small contact angle, small membrane-bound organelles can be fully adsorbed by large condensates, and small condensates may bend the membrane if the lipid bilayer is flexible enough. On the other hand, even in the case of low wettability (i.e., high contact angle), a point-like contact can be maintained through specific anchoring by protein–lipid nanodomains embedded in the membrane. The consequence of these interactions – whether the membrane-bound organelle sticks to the condensate, stays in the condensate or separates from the condensate – depends on the regulatory factors and the specific biological context.

Mechanistically, dipping contacts explain how the activity of biomolecular condensates in the cytosol affects protein–lipid organization and dynamics in the membranes, and vice versa. A molecule that comes to the interface might be in equilibrium with the surrounding phase or it might become trapped (Botto et al., 2012; Bradley et al., 2017), never coming off the interface unless another molecule displaces it. In line with this, at the interface of liquid condensates and membrane-bound organelles, trapped molecules can exist that stabilize the interface (Mason et al., 2017; Simon et al., 2017) or form condensed, jammed layers of proteins, which might serve as percolating structures (a networking transition enabled by the multivalence of specific interactions) able to accommodate stress (Colombo and Gado, 2014; Zwicker et al., 2015; Pappu et al., 2023). Some of these molecules found at interfaces can jam and buckle (Garbin et al., 2012), forming a skin such as that formed by casein on the surface of hot milk (Sadek et al., 2015).

Characterization of dipping contacts will provide clues about the necessary specificity for intracellular trafficking, signaling and metabolic processes. The framework of dipping contacts focuses on what determines the fate of membraneless and membrane-bound compartments after their initial contact; it fits well with the quantitative models that describe phase transitions at surfaces in the presence of membrane binding as a surface wetting phenomenon (Zhao and Zhang, 2020; Kusumaatmaja et al., 2021; Zhao et al., 2021) (see Box 1) and scenarios where membraneless compartments adhere to surfaces as a form of modifiable scaffold (McSwiggen et al., 2019a; Houser et al., 2022).

Several examples of physiological membraneless condensates that are loaded with membranes can provide further insights into dipping contacts, such as the cluster of SVs at the synaptic bouton (Fig. 1B) (Milovanovic et al., 2018), clusters of vesicles in primed B cells, (Wong et al., 2020), and clusters of vesicles in the early secretory pathway (Fig. 1C) (Pranke et al., 2011; Gallo et al., 2023), Golgi (Rebane et al., 2020; Ziltener et al., 2020) and Balbiani bodies (Boke et al., 2016). In these examples, membrane-bound organelles are active participants of the phase, and their integral proteins and lipids are involved in mediating LLPS. Below, we will focus on a prominent biomolecular condensate, the SV condensate, which clusters hundreds of SVs together with proteins essential for neurotransmission (Sansevrino et al., 2023).

## The synaptic bouton – a multicondensate emulsion packed with membranes

**Fig. 3.**
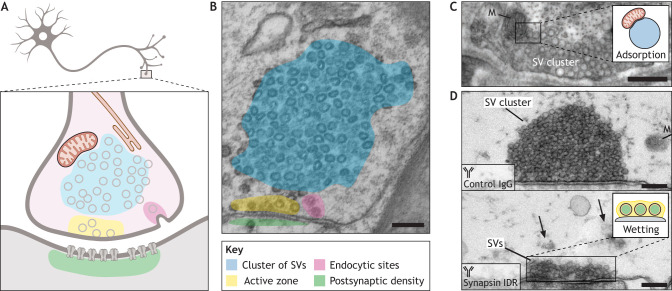
**The synaptic bouton is a multicondensate system packed with membranes.** (A) Schematic illustration of a neuron (top) and a synapse (bottom), emphasizing distinct regions within the pre- and post-synapse. (B) EM image of a synaptic bouton. Different regions of the synapse shown to assemble via LLPS are highlighted. Scale bar: 200 nm. Adapted from Milovanovic and De Camilli (2017) with permission from Elsevier. This image is not published under the terms of the CC-BY license of this article. For permission to reuse, please see Milovanovic and De Camilli (2017). (C) EM image of a murine synapse. Note the mitochondrion (M) juxtaposed to the cluster of SVs. Inset: schematic of a mitochondrion at the surface of a condensate. Scale bar: 250 nm. Image from Rizzoli and Betz (2004). Reprinted with permission from AAAS. This image is not published under the terms of the CC-BY license of this article. For permission to reuse, please see Rizzoli and Betz (2004). (D) EM images of lamprey synapses injected with either a control antibody (top) or an antibody against the IDR of synapsin 1 (bottom). Note that injection of the anti-synapsin 1 antibody leads to dispersion of the SV condensates (arrows), while the SVs adjacent to the presynaptic plasma membrane remain in place, embedded in the active zone condensate. Inset: scheme of a condensate wetting the surface. M, mitochondrion. Scale bars: 250 nm. Adapted from Pechstein et al. (2020), where the images were published under a CC-BY 4.0 license.

A prime example of a multicondensate system is the cell nucleus, where the nucleolus, Cajal bodies, nuclear speckles and sites of transcriptional regulation have all been shown to be formed by LLPS and co-exist in a demixed state (Lafontaine et al., 2021). An emerging example of a multiphase system is the synaptic bouton, which is a key site for neurotransmission (Fig. 3A). During neurotransmission, an action potential (an electric signal) at the presynaptic neuron triggers the release of neurotransmitters (a chemical signal) that will subsequently activate the receptors at the postsynaptic membrane of a receiving neuron (Südhof, 2013, 2021). Coordinated neurotransmission is essential for circuit function and behavior. The synaptic bouton is a specialized site for neurotransmission and a particularly well-suited system to address how membraneless and membrane-bound organelles interact, as compelling evidence suggests that neurotransmitter-containing SVs, which are hallmark structures of the presynapse (Pieribone et al., 1995; Rizzoli and Betz, 2004), are assembled into condensates by LLPS from the surrounding cytosol (Milovanovic and De Camilli, 2017). The SV condensates at the synaptic bouton are highly loaded with the membranes of SVs (Fig. 3B) and represent a reservoir of synaptic proteins that is essential for the SV cycle (Shupliakov, 2009; Denker et al., 2011). In fact, hundreds of SVs form a phase with synapsins (Milovanovic et al., 2018; Pechstein et al., 2020; Park et al., 2021; Hoffmann et al., 2021), which are members of a highly abundant family of synaptic phosphoproteins (De Camilli et al., 1990; Chi et al., 2003). Despite being confined in the separated phase, SVs can move freely and swiftly through the condensate (Joensuu et al., 2016; Hoffmann et al., 2023a) and are recruited to the presynaptic plasma membrane by specific protein and lipid interactions to release their neurotransmitter content (Wu et al., 2014).

The active zone, where SVs fuse with the membrane to release neurotransmitters (Südhof, 2012), is composed of a densely packed network of several proteins that mediate priming and docking of SVs, as well as recruitment of voltage-gated Ca^2+^ channels to the exocytic sites, thereby aligning the active zone to the postsynaptic membrane and mediating synaptic plasticity (Zhai and Bellen, 2004). The major scaffold proteins of the active zone protein complexes are the Rab-3-interacting molecule (RIM) proteins (also known as RIMS proteins), protein unc-13 homolog B (Munc13, also known as UNC13B), RIM-binding proteins (RIM-BPs), α-liprin proteins (also known as PPFIA proteins) and ELKS proteins (also known as ERC proteins), which are conserved in both vertebrates and invertebrates, as well as Piccolo and Bassoon, which are present only in vertebrates (Ackermann et al., 2015). It has been shown that RIM proteins and RIM-BPs (Wu et al., 2019) as well as α-liprin proteins and ELKS proteins (McDonald et al., 2021; Liang et al., 2021) all phase separate into dynamic condensates through multivalent interactions that are mediated by both their intrinsically disordered regions (IDRs) and the interaction between well-defined protein domains and their recognition sequences (for example, SH3 domain–proline-rich motif interactions). These dynamic active zone assemblies can cluster voltage-gated Ca^2+^ channels at the membrane and couple them to the docked SVs (Wu et al., 2019; Emperador-Melero et al., 2021). Similarly, SVs need to be recruited from the SV-containing condensates into the active zone to replenish those vesicles that have fused with the membrane upon neurotransmitter release (Wu et al., 2021); hence, there is a need for an active coupling between these two states. In this context, Piccolo and Bassoon are particularly well suited to recruit SVs from a synapsin–SV condensate as they can form largely extended helices spanning more than 100 nm from the active zone (Glebov et al., 2017). These tethering proteins at the active zone help to assemble the actin cytoskeleton, providing the recruitment tracks for vesicles moving from the SV clusters to the sites of fusion, where neurotransmitter release take place (Gundelfinger et al., 2015).

Both endocytosis and exocytosis are essential for synaptic transmission at the synapses. Upon exocytosis, SVs must be regenerated, which is achieved through two processes that take place at the synapse: clathrin-mediated endocytosis, which occurs in seconds (Saheki and De Camilli, 2012), or ultrafast bulk endocytosis, which operates in milliseconds (Watanabe and Boucrot, 2017). Both of these pathways appear to depend on LLPS. In the case of clathrin-mediated endocytosis, membrane budding is initiated by adaptor proteins, such as Eps15, FCHo1, FCHo2 and intersectin 1, which all form an interconnected network at the plasma membrane to recruit and concentrate downstream endocytic proteins (Day et al., 2021; Kozak and Kaksonen, 2022). In this context, LLPS of the initiator proteins Eps15, FCHo1 and FCHo2 has been shown to be required for productive endocytosis (Day et al., 2021). Eps15, FCHo1 and FCHo2 all contain IDRs and form condensates through weak, multivalent interactions with proteins such as intersectins. Interestingly, intersectin 1 contains five SH3 domains that interact with proline-rich motifs of synapsin 1, enabling recruitment of intersectin 1 into synapsin–SV condensates (Milovanovic et al., 2018). As synapsin–SV condensates are reversible upon depolarization, it is tempting to speculate that SV condensates provide a buffer of endocytic proteins for the fast conversion of synapses from a resting state to an active state (Shupliakov, 2009; Denker et al., 2011; Yoshida et al., 2023).

Several proteins that are involved in clathrin-mediated endocytosis, including dynamins, are also essential for ultrafast endocytosis (Watanabe et al., 2013, 2018). This raises a very important question of how the same factors mediate both slow and fast endocytosis. Recent data suggest that the GTPase dynamin 1, which is responsible for scission of invaginated membranes in endocytosis (Ferguson and De Camilli, 2012), can undergo LLPS with syndapin 1 (also known as PACSIN1), another endocytic effector protein (Imoto et al., 2022). Specifically, a splice variant of dynamin 1 forms condensates at the presynaptic plasma membrane, facilitating the rapid process of ultrafast endocytosis by increasing its local concentration at endocytic sites and thereby bypassing the time-consuming step of recruitment to the site of endocytosis (Imoto et al., 2022). Furthermore, based on analysis of endocytosis in yeast, protein condensates containing the endocytic coat protein Sla1 might provide mechanical work at their interfaces with the plasma membrane and the cytosol, thereby causing membrane invagination (Bergeron-Sandoval et al., 2021). Upon formation of a new interaction surface between the condensate, membrane and cytosol, the adhesion energy of the condensate is released, which can overcome the energy penalty of deforming the membrane and cytosol and leads to invagination of the membrane.

SV condensates, the active zone and endocytic sites all appear to represent distinct biomolecular condensates (Sansevrino et al., 2023), implying that the synaptic bouton is indeed a complex emulsion (Fig. 3A). They also provide a unique biological setting to address a critical question: how is the interface between two or more liquid–liquid phases, or between a liquid phase and a membrane-bound organelle, maintained? It is well established that most membrane-bound organelles are connected to each other via contact sites (Scorrano et al., 2019). There is a great diversity within such membrane contact sites across biology; some, such as the contact sites spanning the mammalian endoplasmic reticulum (ER) and plasma membrane, are as narrow as 19–22 nm, whereas others stretch out over distances of up to 100 nm owing to the presence of long tethering proteins, as is the case for contacts between the ER and mitochondria (Prinz et al., 2020). Membrane contact sites also vary in their stability; they can be highly transient and exist for only a few seconds, as is the case for ER tubules that contact mitochondria during mitochondria division (Friedman et al., 2011), or can last the lifetime of a cell, such as the contacts between sarcoplasmic reticulum and the plasma membrane in muscles (Block et al., 1988). These contact sites are typically functional and mediate, for instance, the transport of essential lipids through lipid trafficking (Lahiri et al., 2015; Reinisch and Prinz, 2021) or the transfer of Ca^2+^ ions to regulate Ca^2+^ homeostasis (Burgoyne et al., 2015). However, the molecular basis of the interaction between membrane-bound organelles (such as the ER and mitochondria) and biomolecular condensates is unknown.

## SV condensates are an ideal system for investigating dipping contacts

In synaptic boutons, entire membrane-bound organelles, such as SVs, have been observed to be fully engulfed in a condensate (Fig. 3B). In addition, mitochondria have been seen to accumulate at the interface of synapsin–SV condensates (Fig. 3C), as well as at active zone condensates in close contact with the presynaptic membrane (Fig. 3D). Experimental data suggest that there is specificity in each of these membrane–condensate interactions. For instance, in *in vitro* reconstituted systems, small liposomes that lack negatively charged phospholipids are not enriched in synapsin condensates (Milovanovic et al., 2018), indicating a role of surface charge in this process. Furthermore, both the headgroups of lipids and the cytosolic tails of membrane proteins contribute to the surface charge of SVs (Bigay and Antonny, 2012). Thus, the interfacial charge of membrane-bound organelles could act as a molecular determinant for partitioning some organelles into the dense phase. For instance, low-affinity electrostatic interactions between positively charged synapsins and the acidic cytosolic tail of synaptophysin are sufficient to generate a condensate of small lipid vesicles when ectopically expressed in non-neuronal cells (Park et al., 2021). Similarly, multiple low-affinity interactions between the glutamate transporter VGLUT-1 (also known as SLC17A7) and endocytic proteins such as endophilin A1 and intersectin 1 all strengthen SV condensates and reduce the exchange of SVs between neighboring boutons (Zhang et al., 2019).

At synaptic boutons, a stereotypic architecture is evident, whereby vesicles recycled by endocytosis are specifically recruited into the liquid phase comprising synapsins and SVs, whereas newly endocytosed vesicles that still contain a clathrin coat are excluded, as are components of the endolysosomal system (Shupliakov et al., 2002). However, occasionally, membrane-bound structures other than SVs, such as small endosomes, dense-core vesicles or plasma membrane infoldings, are present in the SV condensates, whereas large organelles, such as the mitochondria or ER, are completely absent, despite their frequent occurrence and tight packing in synaptic boutons (Rizzoli and Betz, 2004; Wu et al., 2017). Interestingly, the accumulation of mitochondria at the edge of SV condensates, as visualized using electron microscopy (EM), is highly reminiscent of them dipping into an SV phase (Fig. 3C). Here, the downstream fate of the membrane-bound organelles – whether they stay associated with the condensate or dissociate from it – will depend on the specificity and avidity of the interaction between the liquid phase and protein–lipid domains within the membrane-bound organelles (Fig. 2A).

The roles of the interfaces are emerging as a critical feature of biomolecular condensates. Theoretical modeling suggests that ensembles of molecules within a condensate can rearrange into a distinct conformation at interfaces compared to that at the core of the condensates (Farag et al., 2022); this can lead to unique diffusion properties and local chemical activity, such as accelerated redox reactions (Dai et al., 2023). In this context, SV condensates are also well poised for investigations of interfacial properties. For example, synapsin 1 molecules at the interface of SV condensates have a pronounced confinement (Hoffmann et al., 2023a) and can even lead to an accumulation of charge, forming an electrical double layer (Hoffmann et al., 2023b) – a property classically considered to be a feature of membranes. As synapses undergo reversible alterations in ion concentrations during repeated rounds of neurotransmission (Wiegert et al., 2017), coupled to global phosphorylation changes (Kohansal-Nodehi et al., 2016), the synaptic boutons offer an opportunity to identify the principles regulating interactions between condensates and membranes in cell physiology and how a failure to regulate condensate–membrane interactions could yield pathology.

## Organelle accumulation within inclusions in neurodegenerative diseases – the result of disrupted dipping contacts?

**Fig. 4.**
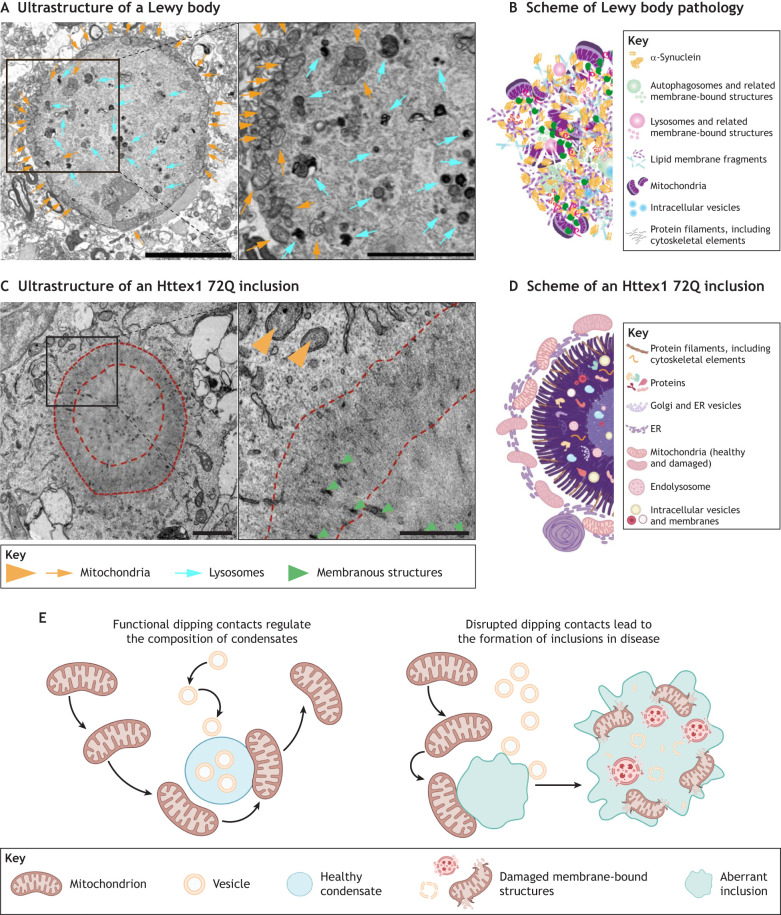
**Aberrant condensates trap organelles and form insoluble inclusions, a hallmark of cellular pathology in neurodegenerative diseases.** (A) Lewy body ultrastructure. Serial block-face scanning EM images of an inclusion from an individual with Lewy body pathology. The entire Lewy body is shown on the left, and an enlarged view of the boxed region is shown on the right. Orange arrows point to accumulated mitochondria at the periphery, cyan arrows point to components of the endolysosomal system. Scale bars: left, 5 µm; right, 2 µm. (B) Schematic illustration of the Lewy body inclusion as a medley of proteins and organelles. A and B are adapted from Shahmoradian et al. (2019). Reproduced with permission from Springer Nature. These images are not published under the terms of the CC-BY license of this article. For permission to reuse, please see Shahmoradian et al. (2019). (C) EM images of a pathological inclusion formed by huntingtin exon 1 with 72 glutamine repeats (Httex1 72Q). Left: the entire inclusion. Right: enlarged view of the boxed region. The shell and the core of the inclusion are delimited by dashed lines. Internalized membranous structures and the mitochondria are indicated by green and orange arrowheads, respectively. Scale bars: left, 1 µm; right, 500 nm. (D) Schematic illustration of an Httex1 72Q inclusion and surrounding organelles. The membrane-bound organelles are depicted to be both trapped in the protein core and accumulated at the surface of the pathological inclusion. C and D are adapted from Riguet et al. (2021), where the images were published under a CC-BY 4.0 license. (E) A model of functional dipping contacts in healthy cells (left) and disease (right). Functional dipping contacts might contribute to the specificity of condensates, regulating which organelles associate with the condensates. This process might be disrupted in pathological conditions, leading to the formation of insoluble medleys of misfolded proteins and disrupted membrane-bound organelles.

Insoluble inclusions are a hallmark of many diseases, including neurodegenerative diseases (Wilson et al., 2023). These inclusions are often formed intracellularly, leading to the disruption of organelle trafficking. A prominent example of this process is the formation of Lewy bodies (LBs), protein deposits that mainly consist of α-synuclein and are a characteristic feature of a family of diseases referred to as synucleinopathies (Wakabayashi et al., 2013). LBs impair neuron function, causing cognitive and motor decline (Fares et al., 2021). LBs exhibit a striking morphological similarity to the inclusions found in Huntington's disease, another debilitating neurodegenerative disease, which contain the polyglutamine-rich protein huntingtin as the main component (Reiner et al., 2011). Recent examples of postmortem human brain tissues of individuals with Lewy body dementia (Shahmoradian et al., 2019), as well as neurons expressing mutated huntingtin proteins (Riguet et al., 2021), show that these aggregates are in fact a crowded medley of vesicular structures (Fig. 4A–D). The components of these medleys – mitochondria, SVs, small endosomes, and synaptic proteins including α-synuclein and huntingtin – are all reminiscent of synaptic components, but their association is completely disrupted, resulting in insoluble inclusions (Fig. 4E).

For several proteins involved in the formation of pathological inclusions [for example, FUS, TDP-43 (also known as TARDBP) and huntingtin], an aberrant phase separation has been shown to result in their aggregation (Patel et al., 2015; Peskett et al., 2018). In the disease state, the cores of these inclusions often contain staggered β-sheet fibrils (Guerrero-Ferreira et al., 2018; Fitzpatrick and Saibil, 2019). Recent *in vitro* analysis of condensates suggests that the interfaces between condensates and surrounding structures could act as local hot spots for protein oligomerization and the formation of fibrils (Shen et al., 2023). This is aligned with data from humans and cellular models revealing that organelles appear to be trapped at the interface of protein deposits (Fig. 4A–D), which has prompted investigations to identify the molecular determinants regulating this adsorption. Although functional dipping contacts can play a role in determining the specificity of condensates, dictating which organelles they interact with, this mechanism might become impaired in pathological conditions, resulting in the creation of insoluble medleys of misfolded proteins and disrupted membrane-bound organelles (Fig. 4E). The framework of dipping contacts between membraneless organelles and membranes thus allows us to consider these diseases as a change in the physicochemical state of the condensates, rather than merely a consequence of pathogenic variants affecting a single component of a particular biochemical pathway.

## Conclusions

As outlined here, characterization of the interface between biomolecular condensates and membranes is critical for understanding how the interface of condensates is coupled to biochemical processes occurring in the membranes (Rouches et al., 2021) and is thus important for signal transduction and intracellular trafficking. Here, we have presented a framework of dipping contacts for investigating the possible interactions between condensates and membranes that can operate on a range of timescales, from milliseconds to seconds in the case of signal transduction, over minutes to hours in intracellular trafficking, to days or even years in the case of, for instance, the storage of mitochondria in Balbiani bodies. As such, dipping contacts exert important cellular functions.

For example, keeping a reserve pool of SVs in a liquid phase allows for their local accumulation and a swift recruitment to the synaptic membrane for fusion upon sustained, prolonged release (Joensuu et al., 2016). Indeed, it has been shown that disrupting the multivalent interactions between proteins within SV condensates disrupts the exchange of vesicles between neighboring synapses (Zhang et al., 2019). Similarly, we and others have shown that the balance of the molar ratios between synaptic proteins, such as synapsins and α-synuclein, and SVs directly impacts the mesoscale organization of the SV condensates, from dispersed, over condensed but mobile, to tightly packed SVs within the dense phase (Vargas et al., 2017; Hoffmann et al., 2021). Thus, it is becoming increasingly clear that the presence of IDRs in membrane-associated proteins has an essential role in balancing the motility of SVs with their accumulation at the synapse (Hoffmann et al., 2023a). Dipping contacts might occur at the interface between the reserve pool of SVs and the active zone, where the proteinaceous phase of the active zone is in contact with the SV phase (Wu et al., 2021), specifically recruiting SVs from the reserve pool to the presynaptic plasma membrane to prime them for fusion upon depolarization (Ogunmowo et al., 2023 preprint). Such dipping contacts could regulate the size and number of release sites in a process similar to how protein nanoclusters at the interface of RNA granules regulate the distribution of these structures in oocyte development (Folkmann et al., 2021; Hoffmann et al., 2022).

In addition to the classical toolbox for investigating condensates and membranes (for a brief overview, see Box 2), the characterization of dipping contacts requires several additional considerations. One example is the development of lipid probes that would reliably report the lipid fluidity or the enrichment of specific lipid species at the contacts between condensates and membranes (Wang et al., 2023). Another aspect is the necessary advancement in crosslinking chemistry that would allow reliable capture of these transient but specific interactions in living systems (Qin et al., 2021). These approaches could be coupled to ultrastructural methods, such as soft X-ray tomography or correlative light–electron microscopy (Weinhardt et al., 2018; Goetz and Mahamid, 2020), providing a label-free approach to map dipping contacts in cells.

**Box 2. A brief overview of approaches for studying condensate–membrane interactions**

The methods to quantify condensate–membrane interactions build upon a multitude of approaches classically used to study membranes and associated protein scaffolds. In particular, minimal *in vitro* reconstitution systems with recombinant proteins, RNA and model lipid membranes (for example, supported lipid bilayers, liposomes of varying size, giant unilamellar vesicles) (Sezgin and Schwille, 2012) are suitable to identify the minimal components and characterize phase behaviors. Moreover, time-resolved and super-resolution microscopy are often the methods of choice for analyses both *in vitro* and in cells (for example, single-molecule tracking, fluorescence recovery after photobleaching, fluorescence lifetime imaging and fluorescence correlation spectroscopy) (Ghosh et al., 2017; Lelek et al., 2021).

In recent years, a range of biophysical techniques for characterizing the material properties of condensates has emerged, including modified applications of optical tweezers, passive microrheology and micropipette aspiration, to name a few (Alshareedah et al., 2021; Wang et al., 2021; Kar et al., 2022). All of these assessments are further strengthened by genetic analyses in model organisms (such as knockout and rescue experiments), which aim to tease out the functions of molecules undergoing LLPS. Although none of these approaches alone is sufficient for an in-depth characterization of condensates, a combination of these methods can allow assessment of the relationship between cause and effect for interactions between condensates and membranes. These experimental approaches are ideally suited to be complemented with computational modeling and simulations (Farag et al., 2022; Zeng and Pappu, 2023), especially as coarse-grain simulations of membranes have already been successfully used to better understand membrane trafficking (Risselada and Grubmüller, 2021; Souza et al., 2021).

For more information on the necessary considerations when evaluating phase separation in cell biology and the analysis of protein–lipid domains in membranes, we refer the reader to a selection of recent publications (Alberti et al., 2019; Ganzinger and Schwille, 2019; Levental et al., 2020; McSwiggen et al., 2019b; Taylor et al., 2019; Musacchio, 2022; Mittag and Pappu, 2022).

Here, we have used synaptic boutons as an experimental model to build the framework of dipping contacts, providing a foundation for exploring a wide range of processes. These processes include intracellular patterning during development, the assembly of viral particles and trafficking of RNA granules, emphasizing that the principles underlying the interactions of membraneless and membrane-bound organelles are important across biological systems.
